# Root Cause Analysis: Unraveling Common Laboratory Challenges

**DOI:** 10.7759/cureus.53393

**Published:** 2024-02-01

**Authors:** Dharini Srinivasaragavan, Karthikeyan Ramalingam, Pratibha Ramani

**Affiliations:** 1 Oral Pathology and Microbiology, Saveetha Dental College and Hospitals, Saveetha Institute of Medical and Technical Sciences, Saveetha University, Chennai, IND

**Keywords:** laboratory error, errors, outcome analysis, oral pathology laboratory, quality improvement, root cause analysis, root cause, pathology, laboratory, fishbone

## Abstract

Diverse errors occur in a pathology laboratory and manual mistakes are the most common. There are various advancements to replace manual procedures with digitized automation techniques. Guidelines and protocols are available to run a standard pathology laboratory. But, even with such attempts to reinforce and strengthen the protocols, the complete elimination of errors is yet not possible. Root cause analysis (RCA) is the best way forward to develop an error-free laboratory, In this review, the importance of RCA, common errors taking place in laboratories, methods to carry out RCA, and its effectiveness are discussed in detail. The review also highlights the potential of RCA to provide long-term quality improvement and efficient laboratory management.

## Introduction and background

**Figure 1:**
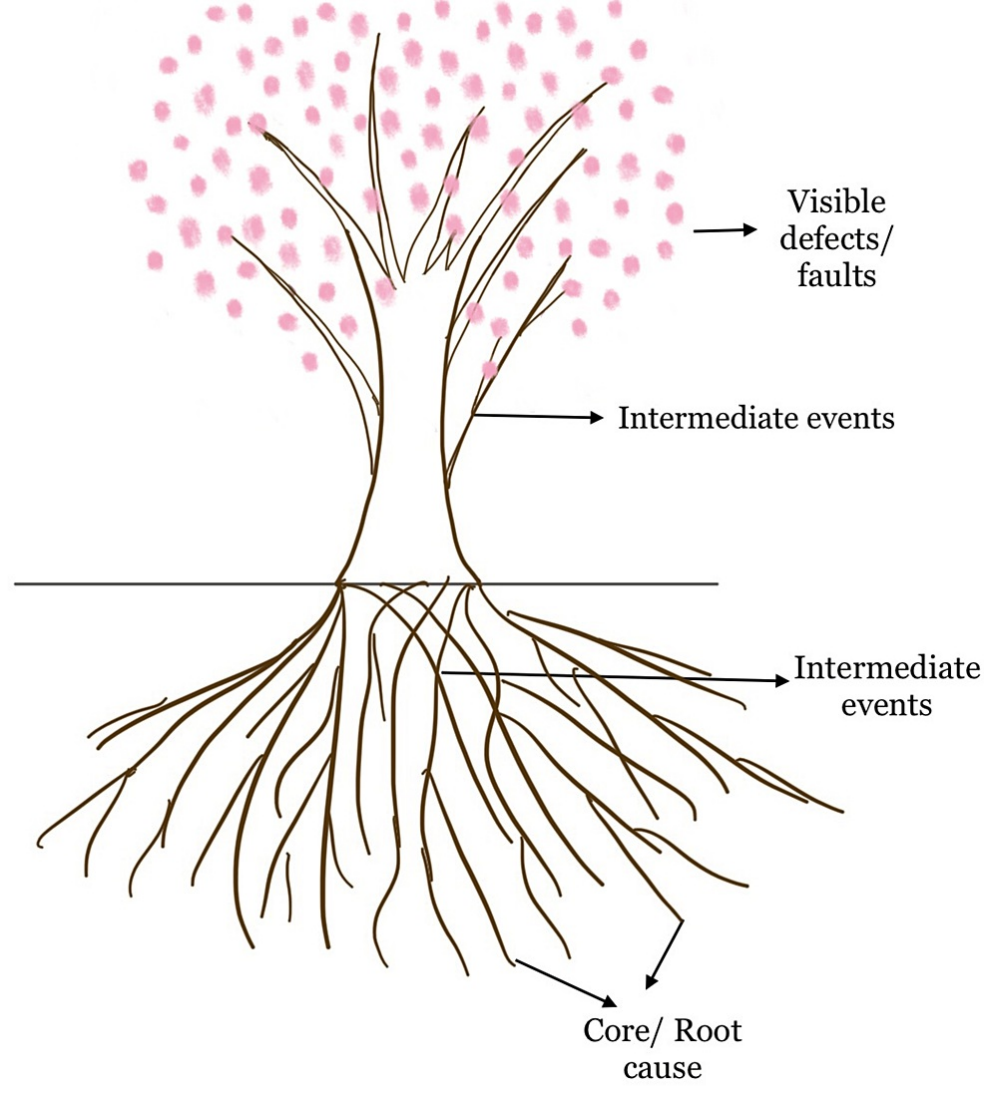
Representation Diagrammatic representation of root cause analysis (Image credit - Dharini Srinivasaragavan, Karthikeyan Ramalingam)

Pathologists must have expertise in lab management in addition to diagnostic skills, as labs are now considered to be one of the highest sources of revenue [1]. Laboratory management has also become an essential aspect, especially with the advent of accreditation. All the activities involved in the lab procedures influence the overall management of a pathology lab [2]. The system starts with receiving samples, recording all the initial patient details followed by processing and carrying out the tests, and ends with reporting. Hence an appropriate design of the lab management system (LMS) ensures that every demanding requirement in the daily routine of handling samples is fulfilled. The advantages of an appropriate LMS involve expansion of business, increased sample testing, and thus eventually fostering high revenues [2, 3]. One promising approach that aids in maintaining a standard lab management system is root cause analysis (RCA). RCA identifies the various reasons for the outcome and helps to initiate corrective action (Figure 1).

Root cause analysis

Root cause analysis (RCA) states that there could be one or more causes for every event. RCA plays an important role in avoiding the cascading effect of multiple incidents (apparent/unobserved events) that lead to a final defect (critical/observable event) which is more devastating [4]. Hence RCA is a systematic approach that focuses on finding flaws that could be refined for better performance and predictable outcomes. 

An RCA team is assembled to examine the observed event and to determine required changes on a systematic basis. Analyzing and defining the issue of concern is the primary step in RCA following which listing and assessment of all contributing factors are carried out. A comprehensive data collection of each step is helpful for better evaluation and to ascertain their interrelationships. During the RCA process, the team must consider intermediate actions constituting strategies to immediately improve the process and to prevent repeat events. Such actions must be consulted with the higher authorities to emphasize the identification of specific targets and achieve significant risk reduction [5]. Thus, RCA should be clear and exact, providing proper in-depth information for any issue of concern (Figure 1).

## Review

**Figure 2:**
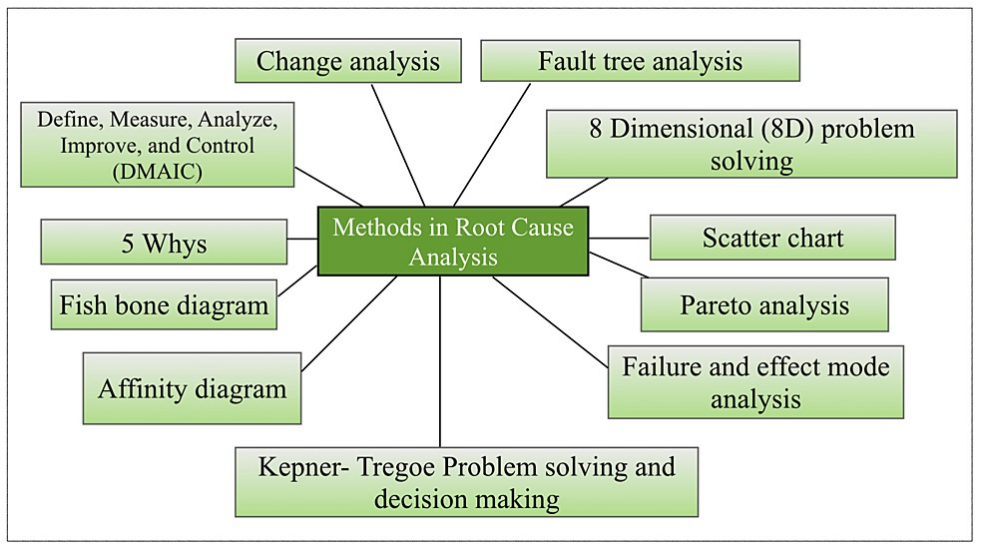
Methods Methodologies reported in the literature to carry out root cause analysis (Image credit - Dharini Srinivasaragavan, Karthikeyan Ramalingam)

Root cause analysis (RCA) states that there can be one or more causes for every event [4]. This kind of cascading effect leads to a final defect that is appreciably more devastating than the apparent faults [5]. There are various approaches to carry out this analysis (Figure 2).

Five whys

Asking why five times for any issue can help in identifying the bottom of the problem [6]. For instance, in a pathology laboratory, the possible five whys and how the reasons for unsatisfied hematoxylin and eosin (H&E) stained sections can be identified are as follows: “Why was the staining unsatisfactory? The staining procedure needs to be improved. Why was the staining protocol not followed? The staining protocol was followed by the technician but the stains used were not in good condition. Why were the stains not in good condition? The stains might have been contaminated due to incomplete or improper washing during the staining procedures. Why were the stains not changed periodically? The new stains were not available in the laboratory. Why was the laboratory stock insufficient? The reagents were not ordered from the store as per schedule”.

Thus, RCA of a problem of unsatisfactory staining was identified as insufficient reagents. A proper cutoff value for each laboratory reagent could be created, a weekly audit of available stock should be performed, and the deficiency should be noted with the immediate purchase of required reagents. Thus, RCA could be utilized to avoid this problem in the future. 

Fishbone diagram

Fishbone analysis, also called cause and effect diagram or Ishikawa diagram, is a visualization tool more like a mind map. It is called the Ishikawa diagram since it was invented by Dr. Karur Ishikawa, a Japanese quality control expert. It helps to find almost all the possible causes for a problem or when we plan for action by including thoughts and ideas from team members [5, 6].

The concerned issue must be written on the right side of the whiteboard. An arrow should be drawn ending to the concerned issue. Preferably six major categories that led to the issue must be written as primary branches. Each major category should list all the possible causes under it. Thus, the fishbone should be filled with multiple possibilities that could cause the concerned issue [6, 7]. By following the above-mentioned procedure, the root cause of a problem could be identified and resolved.

Other methods

A scatter chart employs dots to depict numeric variable values, facilitating the observation of relationships between these variables. It is valuable for identifying correlations, whether positive, negative, or nonexistent, among the variables under consideration. Scatter charts find applications in quality assessment, root cause analysis, and decision-making. It includes correlation analysis, It is a versatile tool in quality improvement, providing a visual representation of data that facilitates analysis, decision-making, identification of trends and patterns, root cause analysis, process optimization, performance measurement, support for decision-making, continuous monitoring of processes, and quality control. It aids in identifying areas for improvement and supports evidence-based interventions to enhance overall product or service quality.

In failure mode and effect analysis (FMEA), the initial step involves identifying failure modes, followed by an effect analysis where the consequences of each cause are analyzed. This process helps uncover potential pitfalls and assess the impact of proposed changes on system design. The key concepts in FMEA include severity (S) - a measure of the seriousness of the consequences of a failure. It is typically rated on a scale from 1 to 10, with higher values indicating more severe consequences. Occurrence (O) - an assessment of the likelihood or frequency of a failure mode occurring. It is also rated on a scale from 1 to 10, with higher values indicating a higher likelihood. Detection (D) - a measure of the likelihood that the failure mode will be detected before reaching the customer. It is also rated on a scale from 1 to 10, with higher values indicating a lower likelihood of detection. Risk priority number (RPN) - calculated by multiplying severity, occurrence, and detection ratings (RPN = S * O * D). Higher RPN values indicate higher-priority failure modes. Thus, FMEA is a valuable tool for proactive risk management, fostering a culture of continuous improvement and helping to enhance the reliability and quality of the processes, products, and systems.

In fault tree analysis (FTA), the problem is visualized as a tree, with probable causes listed as branches. The root cause is identified by examining the causes and their occurrences. The steps in FTA are: 1) Define the top event - clearly define the undesired event or failure that is the primary focus of the analysis. This is the "top event." 2) Identify basic events - identify the basic events that could contribute to the top event. These are the root causes or factors that may lead to the failure. 3) Construct the fault tree - use logic gates to represent the relationships between events. 'AND' gates indicate that all input events must occur for the output event to occur, 'OR' gates indicate that at least one input event must occur, and 'NOT' gates indicate negation. 4) Assign probabilities - assign probabilities to basic events to represent the likelihood of their occurrence. These probabilities are used to calculate the overall probability of the top event. 5) Analyze and evaluate - analyze the fault tree to determine the probability of the top event occurring. Evaluate the critical paths and contributing factors. 6) Mitigation and prevention - identify potential measures or actions to mitigate the risk of the top event. These measures could involve improving system design, implementing safeguards, or enhancing operational procedures. 7) Documentation and communication - document the fault tree analysis, including the identified events, logic gates, probabilities, and conclusions. Communicate the findings to relevant stakeholders. Thus FTA is a powerful tool for understanding and managing risks associated with complex systems. It helps to proactively address potential failures and develop strategies to enhance the reliability and safety of their systems.

Pareto analysis, named after the Italian economist Vilfredo Pareto, is a technique used to prioritize and focus efforts on the most significant factors contributing to a problem or goal. It is based on the principle that a small number of causes (the "vital few") usually account for the majority of the effects, while a larger number of causes (the "trivial many") contribute less significantly. Pareto analysis graphically separates various aspects of a problem, aiding in directing improvement efforts effectively. The Pareto chart, incorporating both a line graph and a bar chart, highlights significant issues based on cumulative effects.

Define, Measure, Analyze, Improve, and Control (DMAIC) is a structured approach that utilizes data-driven decision-making, root cause analysis, continuous improvement, and standardization. DMAIC investigates the reasons for an issue from its definition to controlling all root causes. Its role in the overall success of Six Sigma is crucial, requiring the identification of root causes and the development of solutions. DMAIC involves defining and measuring the issue, analyzing collected data to understand root causes, improving actions, and implementing control measures.

The eight dimensional (8D) problem-solving process is a structured, team-oriented approach widely used in various industries to address and resolve complex problems. The goal of the 8D process is to identify the root cause of a problem, develop effective corrective actions, and prevent the recurrence of the issue. Each "D" represents a step in the process. In 8D problem-solving, the root cause is investigated through a meeting using statistical data. The process involves team formation, defining the problem, temporary action, root cause analysis, permanent corrective action, validation, prevention, and closure. The 8D method is a team-oriented approach combining tools from various disciplines to solve critical production problems.

Change analysis in quality control refers to the process of observing, analyzing, and documenting changes in a system, process, or product over a period of time. This method involves a careful and systematic examination of variations or alterations to identify potential causes and effects. Change analysis is crucial for ensuring that modifications, whether intentional or unintentional, do not negatively impact the quality and performance of the system. Change analysis emphasizes observing changes over a longer period to identify potential causes of issues. It is considered an easy and effective approach. Kenner-Tregoe problem-solving and decision-making, akin to DMAIC, involves situation analysis, problem analysis, solution analysis, and potential problem analysis. This systematic approach helps address causes and significantly improves working conditions [7, 8].

Common challenges

**Figure 3:**
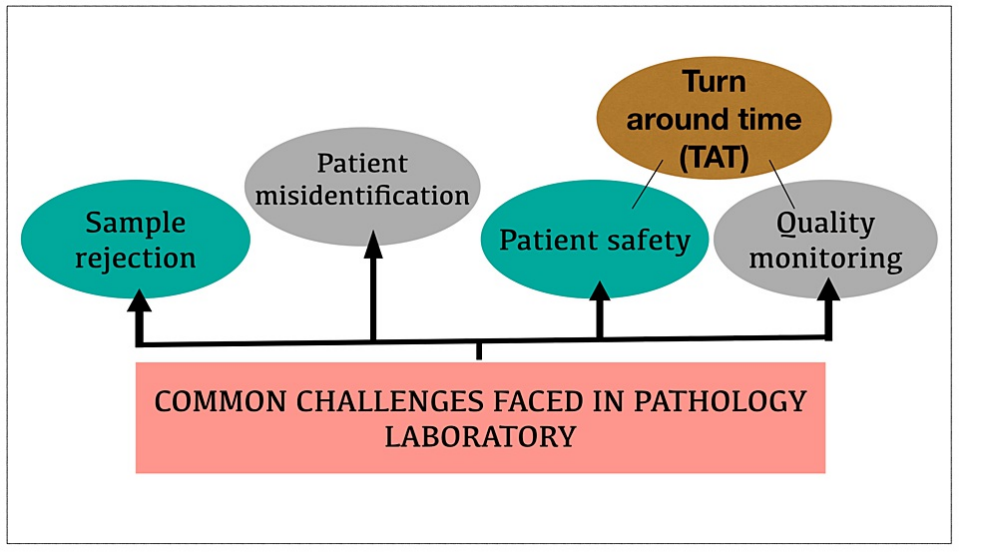
Common challenges Pictorial representation of commonly encountered challenges in pathology laboratory (Image credit - Dharini Srinivasaragavan, Karthikeyan Ramalingam)

Three crucial aspects where errors occur frequently in a pathology laboratory are represented in Figure 3. 

The common challenges encountered in a pathology laboratory like sample rejections, patient misidentification, quality monitoring turnaround time, and safety are discussed below.

Sample Rejections

Laboratory test results serve as the backbone for clinical decision-making as the precision of results has a direct impact on the patient’s management [9]. Broadly, there are three phases in the lab investigation which include the pre-analytical phase, analytical phase, and post-analytical phase. With advancements in the analytical phase and improvements in the quality maintenance protocols, analytical errors were comparatively less common among the three phases [10]. On the other hand, 80 to 93% of the errors were attributed to the pre-analytical phase [11]. This is because the processes are still manual and often subjected to repeat sampling which ultimately burdens the patients. Human errors constitute about 82.6% while only 4.3% are technical. A study by Chavan et al. reported 174 rejected out of 48,889 samples. RCA carried out in this study revealed that most of the sample rejections were identified in the intensive care unit (ICU), hematolymphoid cancer unit, and wards. Some of the potential causes identified were hemolyzed samples, clotted samples, labeling errors, and sample contamination with intravenous fluids. Following four years of training programs, these errors were reduced significantly. Favorable outcomes of the training programs depend on relevant training design, the ability of the trainee to understand, the assessment of the trainee, and the trainee’s ability to apply the skills learned in their workplace. In addition, periodic conduction of retraining was also important [12]. It was also recommended to incorporate activities in the training design and share the results. This brings prominent changes in the trainee’s behavior as the results stimulate a sense of contest [13]. 

Patient Misidentification

Many laboratories use direct label printers but manual data entry/recording is still performed within a few stages such as embedding, sectioning, and staining. Printed labels are then utilized for permanent identification and storage. Misidentification can happen during this process [14]. RCA on misidentification found the following potential causes: slide-labeling in batches, interruption and diversion during labeling, misinformation from manual data entry, misplaced and lost specimens, reporting into the incorrect patient medical record, and small fonts that make visualization difficult [15]. With careful consideration of each of these possibilities, the error rate could be reduced. The drawbacks of manual writing can be avoided by the implementation of automatic barcode systems for sample handling [16]. 

Quality Monitoring and Turnaround Time

Quality monitoring (QM) is essential in a lab to acknowledge errors and make continuous efforts to minimize them to maintain a standard quality. The majority of findings from institutional laboratories in India showed compromised sensitivity and specificity along with delayed laboratory reports [17]. According to the current principles, pathology labs must have a structured, systematic, and organized QM procedure focusing on enhancing patient safety, minimizing error, and guaranteeing prompt report delivery. Additionally, improved performance could be facilitated by quality assurance that incorporated both internal and external quality monitoring [18]. Sandra et al. listed the possible errors occurring in a lab setup and discussed the steps undertaken to minimize them [19]. The most notable root causes of the failures of immunohistochemistry were identified to be a lack of standardization procedures and inconsistent monitoring of the qualitative and quantitative parameters [20]. 

Turnaround time (TAT) is another crucial metric for assessing the quality of a lab. TAT indicates the amount of time required for a laboratory test from order receipt to result generation for the clinician or patient [21, 22]. Efficient clinical workflow is mandatory to maintain TAT for every sample. RCA could be used to identify various factors influencing TAT including technician's availability, pathologist’s experience, automation, infrastructure, and continuous training of laboratory personnel [23]. Storage of samples and reagents influences delayed reporting. Improperly stored or expired reagents can lead to a large number of highly compromised false positive and negative results. It results in repeat biopsies [24]. In addition, comprehensive management of documents is also crucial. Equipment calibration and maintenance must be done regularly. Each laboratory must have its standard operating procedures (SOP) consisting of lists with elaborative instructions for every procedure that the lab performs [25]. Khan et al. emphasized the use of simple guidelines like mentioning the laboratory’s directions on the outpatient department (OPD) card could substantially reduce the TAT and subsequently improve patient satisfaction [26]. 

RCA on the most frequent errors occurring in anesthesia cases of complex procedures were found to be fine skills of the experts and knowledge deficiencies, poor time management, lack of communication, unfamiliarity with the equipment, and drug-related errors [27]. RCA has also been used to identify the causes of myocardial infarction deaths [28]. In addition, in terms of frozen sections, the central placement of the pathology department is close to the operating room and improved communication with surgeons had better outcomes [29]. 

Recommendations

To improve the overall quality of a laboratory, an RCA team/group should be set up to review the feedback and conduct error audits at periodic intervals. It will be highly effective to implement procedural and behavioral changes resulting in error reduction. Implementation of several simple techniques mentioned so far in each step and discussion with the reporting pathologists for appropriate lab management and development can bring significant changes.

## Conclusions

Root cause analysis has been an eye-opener that aids in revealing new insights in almost all disciplines. Especially in laboratories with pertinent quality assurance practices and error detection followed by system design change, the performance can be improved. Numerous factors need to be considered including well-defined diagnostic criteria, precise instrumentations, access to clinical data, appropriate communication between clinicians and pathologists, appropriate sampling and handling, management of excessive workloads, inexperienced pathologists, shortage of skilled technicians, complex cases including specialized pathologists, and so forth. Hence, for any field, root cause analysis is very helpful for long-term consistent improvement in any profession.
